# Interindividual differences in attentional vulnerability moderate cognitive performance during sleep restriction and subsequent recovery in healthy young men

**DOI:** 10.1038/s41598-021-95884-w

**Published:** 2021-09-27

**Authors:** Gina Marie Mathew, Stephen M. Strayer, Kelly M. Ness, Margeaux M. Schade, Nicole G. Nahmod, Orfeu M. Buxton, Anne-Marie Chang

**Affiliations:** 1 Department of Biobehavioral Health, College of Health and Human Development, Pennsylvania State University, 219 Biobehavioral Health Bldg, University Park, PA 16802 USA; 2 College of Nursing, Pennsylvania State University, University Park, PA USA; 3 Present Address: Department of Medicine, Division of Metabolism, Endocrinology, and Nutrition, University of Washington, Seattle, WA USA; 4 Present Address: Department of Medical Science, Arcadia University, College of Health Sciences, Glenside, PA USA

**Keywords:** Sleep deprivation, Attention, Working memory

## Abstract

We investigated whether interindividual attentional vulnerability moderates performance on domain-specific cognitive tasks during sleep restriction (SR) and subsequent recovery sleep. Fifteen healthy men (*M* ± *SD*, 22.3 ± 2.8 years) were exposed to three nights of baseline, five nights of 5-h time in bed SR, and two nights of recovery sleep. Participants completed tasks assessing working memory, visuospatial processing, and processing speed approximately every two hours during wake. Analyses examined performance across SR and recovery (linear predictor *day* or quadratic predictor *day*^2^) moderated by attentional vulnerability per participant (difference between mean psychomotor vigilance task lapses after the fifth SR night versus the last baseline night). For significant interactions between *day*/*day*^2^ and *vulnerability*, we investigated the effect of *day*/*day*^2^ at 1 *SD* below (less vulnerable level) and above (more vulnerable level) the mean of attentional vulnerability (*N* = 15 in all analyses). Working memory accuracy and speed on the Fractal 2-Back and visuospatial processing speed and efficiency on the Line Orientation Task improved across the entire study at the less vulnerable level (mean − 1*SD*) but not the more vulnerable level (mean + 1*SD*). Therefore, vulnerability to attentional lapses after SR is a marker of susceptibility to working memory and visuospatial processing impairment during SR and subsequent recovery.

## Introduction

Short sleep duration is associated with impaired performance, with consequences ranging from reductions in workplace productivity^1^ to increased risk for motor vehicle accidents^2,3^. Vigilance, or the ability to attend to relevant stimuli, is impaired by chronic sleep restriction^4–11^, with decrements persisting even after subsequent recovery sleep^4,7^. However, susceptibility to performance decrements varies widely among persons after both chronic sleep restriction and total sleep deprivation^12^. Lapses of attention, or reaction times (RTs) ≥ 500 ms, exhibit progressively increasing standard deviations across persons over time during total sleep deprivation^13,14^, and interindividual vulnerability to total sleep deprivation explains 58% of overall variance in lapses on the psychomotor vigilance task (PVT)^15^. Individuals who exhibit more lapses during sleep loss are considered “vulnerable” to the effects of sleep loss on attentional performance compared to their more “resistant” counterparts^14,16^. Interindividual vulnerability to impaired vigilance during sleep loss may be driven by neurological mechanisms^17^, such as reduced activity in task-related regions within the frontoparietal network^18^ and extrastriate visual cortex^19^, reduced connectivity between frontoparietal and thalamic regions^19,20^, and/or insufficient suppression of the default mode network^21^ during task completion in vulnerable compared to resistant individuals during sleep loss.

Some evidence suggests that sleep loss (whether total or partial) impairs performance in other cognitive domains, such as working memory^22,23^, visuospatial processing^24,25^, and processing speed^10,26^. After two nights of sleep restricted to 4 h’ time in bed (TIB) per night, participants recalled fewer word pairs compared to an 8-h TIB condition^22^, and total sleep deprivation resulted in slower RT and impaired classification accuracy on an *n*-back task of working memory^23^. Some studies have found that sleep deprivation impairs visuospatial processing by, for example, reducing accuracy in tasks of ball tracking^24^ and saccadic eye movements^25^. Additionally, participants make fewer correct responses on the Digit Symbol Substitution Task (DSST), a test of processing speed, during sleep restriction^10,26^. Other studies suggest that sleep loss does not impair domain-specific performance; for example, studies have found that visuospatial processing in a line orientation task^27^ and working memory in an *n*-back task^28^ are not impaired by total sleep deprivation. A meta-analysis found that tests of simple attention (e.g., the PVT) are more sensitive to the effects of sleep loss than more complex, domain-specific performance^29^.

Similar to vigilance, performance in other cognitive domains during sleep loss demonstrates interindividual differences^30–34^. For example, one study in healthy young men found that some participants exhibited no errors and faster RT on a verbal working memory task after 30 h of total sleep deprivation, whereas others exhibited slower RT and increased errors^31^. In another sample of healthy adults, performance on a different verbal working memory task demonstrated a higher standard deviation among participants after 35 h of total sleep deprivation compared to full rest^30^. After total sleep deprivation of 24 h, throughput on a mathematical processing speed task demonstrated substantial interindividual variation in healthy young adults, with some participants exhibiting “cognitive resistance” and others demonstrating severe impairment^32^. Furthermore, high intraclass correlation coefficients (ICCs) for cognitive performance across multiple sessions of total sleep deprivation indicate that interindividual differences in susceptibility to impaired working memory^14,33^ and processing speed^14^ during sleep loss are reproducible and possibly trait-like.

In some individuals, sleep restriction and total sleep deprivation impair both vigilance^4–11^ and performance in other cognitive domains^10,22–26^. This supports the vigilance hypothesis, which postulates that insufficient sleep induces a global decline in attention^29^. That is, sleep loss affects multiple cognitive domains in a global manner through impaired attention, rather than impairing performance in each domain specifically. A meta-analysis of the impact of total sleep deprivation on both simple attention and more complex domain-specific performance found that performance was impaired across multiple cognitive domains, with the largest effect sizes for tests of simple attention (e.g., the PVT)^29^. The authors posited that deficits in sustained attention may represent the most parsimonious explanation for deficits in more complex cognitive domains during sleep loss. This hypothesis is supported by another meta-analysis that found similar patterns of brain activation (reduced parahippocampal gyrus, bilateral insula, right prefrontal cortex, medial frontal gyrus, and bilateral intraparietal sulcus activity; increased thalamic activity) during total sleep deprivation across both simple and complex attention tasks^35^.

The vigilance hypothesis suggests that those with more attentional deficits during sleep restriction will also exhibit deficits in more complex cognitive tasks, while those who demonstrate fewer attentional deficits will have comparatively unimpaired performance on these tasks. One study that investigated this hypothesis only examined performance on a complex test of selective attention, finding that individuals more vulnerable to attentional lapses after total sleep deprivation were also less accurate compared to resistant participants^36^. To our knowledge, no study has examined whether interindividual differences in attentional vulnerability after several days of sleep restriction moderate the impact of sleep restriction and subsequent recovery sleep on performance in multiple cognitive domains.

In the current study, we investigated whether attentional vulnerability (defined as the change in lapses from the last baseline day to the last sleep restriction day, per participant) during sleep restriction would moderate the impact of sleep restriction and subsequent recovery sleep on working memory, visuospatial processing, and processing speed performance. We hypothesized that those more vulnerable to attentional impairment after five nights of sleep restriction would demonstrate more deficits in tasks assessing other cognitive domains compared to less attentionally vulnerable participants.

## Results

### Demographic information

Two individuals discontinued participation during the study and were excluded from all analyses. The final sample to complete the 11-day inpatient protocol consisted of 15 healthy males, (*mean* ± *SD*) age = 22.3 ± 2.8 years, with an ethnoracial composition of 60% White/Caucasian (*n* = 9), 20% Black/African American (*n* = 3), and 20% Asian (*n* = 3).

### Sleep during pre-study monitoring and inpatient study

All measures examined from actigraphy (sleep onset, midpoint, offset, total sleep time [TST], and maintenance efficiency) differed significantly among habitual, pre-study monitoring, and baseline (conditions (all *p* < .001; see Table S1). Participants provided a minimum of 3 valid nights of actigraphy data each for pre-study monitoring and habitual conditions and 3 valid nights for the baseline condition (first night, the habituation night, was excluded from actigraphy analyses). Sleep timing (onset, midpoint, and offset) became significantly earlier from both habitual to pre-study (onset and midpoint: *p* < .001; offset: *p* = .012) and from pre-study to baseline conditions (onset and midpoint: *p* < .001; offset: *p* = .007). Additionally, TST increased from habitual to pre-study by 0.84 h (*p* < .001), but not from pre-study to baseline (*p* = .160). Sleep maintenance efficiency decreased by 1.4% (i.e., from 88.4% to 87.0%) from habitual to pre-study (*p* = .020), potentially due to the increased TIB, but significantly increased by 2.9% (i.e., from 87.0% to 89.9%) from pre-study to baseline (*p* = .001) and was not significantly different between habitual and baseline (*p* = .119). Therefore, the imposed 10-h pre-study TIB successfully adapted participants to the baseline in-lab sleep schedule. Sleep maintenance efficiency initially decreased during the pre-study sleep monitoring, but rebounded to habitual levels during the baseline condition.

As measured through polysomnography (PSG), we confirmed that a significant difference in TST among conditions was accomplished by the sleep restriction protocol design in linear mixed models (first night excluded), *F* (2, 101) = 937.57, *p* < .001. In pairwise comparisons, TST was shorter during sleep restriction (*M* = 4.76 h, *SEM* = 0.09) compared to baseline (*M* = 8.55 h, *SEM* = 0.12), *t*(101) =  − 32.33, *p* < .001, and longer during recovery (*M* = 9.01 h, *SEM* = 0.12) compared to both baseline, *t*(101) = 3.34, *p* = .003, and sleep restriction, *t*(101) = 36.85, *p* < .001. Of all nights considered (nine nights per participant: two baseline, five sleep restriction, and two recovery; 135 total), 13% were deemed unscorable and excluded from TST analysis.

### Interactions between study day and attentional vulnerability on cognitive performance

The moderator *attentional vulnerability* had a mean of 4.43 (*SD* = 4.66). See Table 1 for interactions between attentional vulnerability (defined as the change in mean PVT lapses between the last baseline day and the last sleep restriction day) and *day* or *day*^2^ (linear or quadratic trajectory for study day, depending on best fit; analyses included last baseline day, five sleep restriction days, and two recovery days) on domain-specific performance (all linear mixed models) and Supplementary Table S2 for comparisons of performance on each sleep restriction and recovery day to the baseline reference day (succeeding the third 10-h TIB sleep period). Supplementary Table S3 displays interactions between attentional vulnerability and *day* or *day*^2^ on performance for factors extracted through principal factor analysis (PFA), and Supplementary Table S4 shows comparisons to the baseline reference day. For significant interactions, the effect of *day* or *day*^2^ on each outcome is shown separately at 1 *SD* below the between-person mean in attentional vulnerability (less vulnerable level) and at 1 *SD* above the mean (more vulnerable level); for non-significant interactions, the effect at the mean is shown. In all analyses, *N* = 15 participants.

**Table 1:** Interactions between day and attentional vulnerability on performance.

Outcome	Predictor	Interaction			By Vulnerability			
		*b *(*SEM*)		ΔR^2^	Level	*b *(*SEM*)		ΔR^2^
**PVT**								
Lapses^§^	Day^2^	− .052***	(.011)	.045	*LV*	− .06	(.07)	.001
					*MV*	− .55***	(.07)	.101
**VOLT**								
Correct responses	Day	− .006	(.007)	.001	*Mean*	− .08**	(.03)	.010
Hits	Day	− .005	(.005)	.001	*Mean*	− .09***	(.02)	.021
Correct rejections	Day	− .002	(.004)	< .001	*Mean*	.01	(.02)	.001
Median RT, correct (ms)	Day	.429	(.577)	.001	*Mean*	− 15.58***	(2.57)	.049
*Response confidence*^a^								
Incorrect responses	Day	.046	(.124)	< .001	*Mean*	1.39**	(.49)	.014
Misses	Day	−.107	(.161)	.001	*Mean*	1.57**	(.60)	.014
False alarms	Day	.142	(.247)	.001	*Mean*	.48	(.95)	.001
**F2B**								
Accuracy^b^	Day	− .056**	(.020)	.011	*LV*	.52***	(.13)	.022
					*MV*	< .01	(.13)	< .001
Sensitivity^b^	Day	− .189**	(.063)	.013	*LV*	2.22***	(.41)	.040
					*MV*	.46	(.41)	.002
Specificity^b§^	Day	− .013	(.016)	.001	*Mean*	− .10	(.07)	.003
Median RT, hits (ms)	Day	.969**	(.353)	.011	*LV*	− 10.48***	(2.28)	.029
					*MV*	− 1.45	(2.28)	.001
**LOT**								
Correct responses	Day^2^	.001	(.006)	< .001	*Mean*	.08**	(.03)	.013
Rotation error^c^ (*M*)	Day^2^	< .001	(.001)	< .001	*Mean*	− .01*	(< .01)	.009
Excess clicks, correct (*M*)	Day^2^	− .005**	(.001)	.023	*LV*	.06***	(.01)	.094
					*MV*	.02	(.01)	.008
Median RT, correct (s)^§^	Day^2^	− .004**	(.001)	.019	*LV*	.02*	(.01)	.060
					*MV*	− .01	(.01)	.011
**DSST**								
Throughput^d§^	Day^2^	.015	(.010)	.006	*Mean*	.20***	(.04)	.031
Median RT, correct (ms)^§^	Day^2^	− .190	(.135)	.003	*Mean*	− 2.35***	(.61)	.020

Vulnerability calculated as difference between within-person mean of psychomotor vigilance task (PVT) lapses on last sleep restriction day versus last baseline day, per person (see Fig. 6). For significant *day* or *day*^2^*vulnerability interactions, the effect of *day* or *day*^2^ at lower vulnerability (LV, less vulnerable) and at higher vulnerability (MV, more vulnerable) levels is shown. LV estimates were obtained by re-centering attentional vulnerability at 1 standard deviation (*SD*) below the mean. MV estimates were obtained by re-centering attentional vulnerability at 1 *SD* above the mean (*N* = 15 in all analyses; refer to Statistical Analyses). For non-significant interactions, the effect of *day* or *day*^2^ at the mean of vulnerability is depicted.

ΔR^2^, change in R^2^ for model term; *b*, unstandardized beta; DSST, Digit Symbol Substitution Task (processing speed)^49^; F2B, Fractal 2-Back (working memory)^39^; LOT, Line Orientation Task (visuospatial processing)^48^; LV, less vulnerable; *M*, mean; MV, more vulnerable; PVT, psychomotor vigilance task (attention)^66,67^; RT, reaction time; s, seconds; *SEM*, standard error of the mean; VOLT, Visual Object Learning Task (working memory)^40^.

^a^Range: 0 (minimally confident) to 100 (maximally confident).

^b^Range: 0 (minimally accurate/sensitive/specific) to 100 (maximally accurate/sensitive/specific).

^c^Range: 0 (no rotation error) to 30 (perpendicular line; highest rotation error).

^d^Number correct per minute.

^§^Model includes *time of day*.

*p < .05; **p < .01; ***p < .001, two-tailed.

#### *PVT lapses* (*vigilance*)

**Figure 1:**
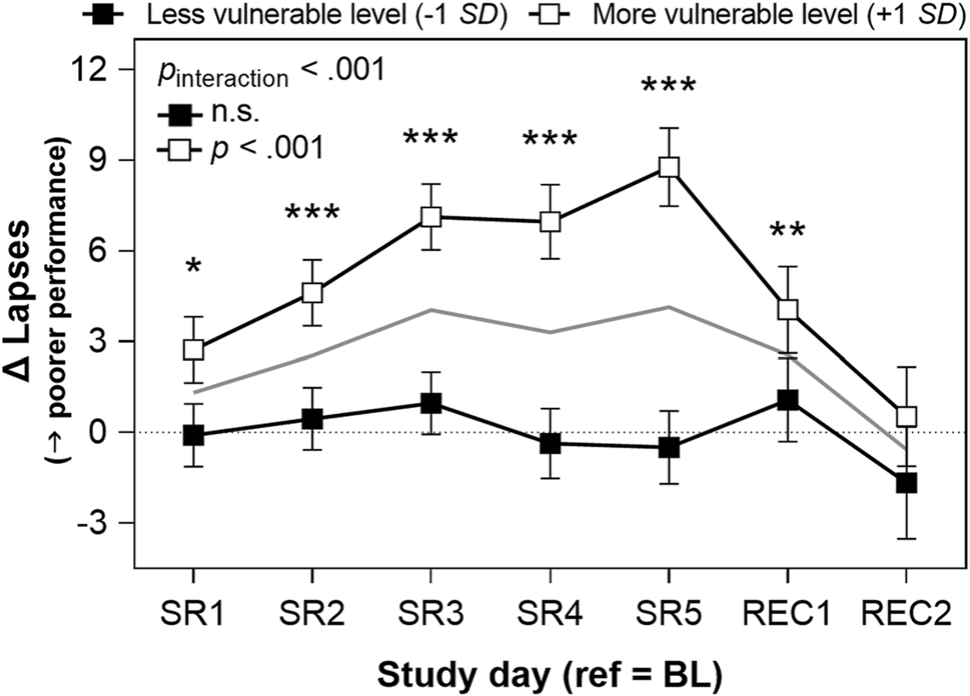
Psychomotor vigilance task (PVT) lapses by vulnerability per participant across sleep restriction (change from baseline). PVT lapses are reaction times ≥ 500 ms. “Less vulnerable level” estimates were obtained by re-centering attentional vulnerability (i.e., difference in mean lapses on last sleep restriction day relative to the last baseline day for each participant) at 1 standard deviation (*SD*) below the mean. “More vulnerable level” estimates were obtained by re-centering attentional vulnerability at 1 *SD* above the mean (*N* = 15 in all analyses; refer to Statistical Analyses). See Fig. 6 for vulnerability level per participant. Change from baseline (BL) values are plotted; horizontal dotted line indicates the baseline reference level (where change is 0). Comparisons to the BL reference point (the day succeeding the third BL night) during sleep restriction (SR) and recovery (REC) day separated by vulnerability level: *p < .05, **p < .01, ***p < .001, two-tailed. Comparisons to baseline not completed at “less vulnerable level” due to lack of significant trajectory (see Table 1, “by vulnerability” column). Performance at the mean of attentional vulnerability is represented by the gray line. Error bars depict standard error of the difference. Values are estimated from linear mixed modeling.

There was a significant interaction between vulnerability and *day*^2^ on PVT lapses (RTs ≥ 500 ms; *p* < .001). At the more vulnerable level (i.e., at 1 *SD* above the mean of vulnerability), PVT lapses changed significantly throughout the protocol in a U-shaped trajectory (*p* < .001; see Fig. 1). This contrasted with the less vulnerable level (i.e., at 1 *SD* below the mean of vulnerability), at which PVT lapses did not significantly change across the protocol (*p* = .400).

#### *Visual Object Learning Task* (*working memory*)

**Figure 2:**
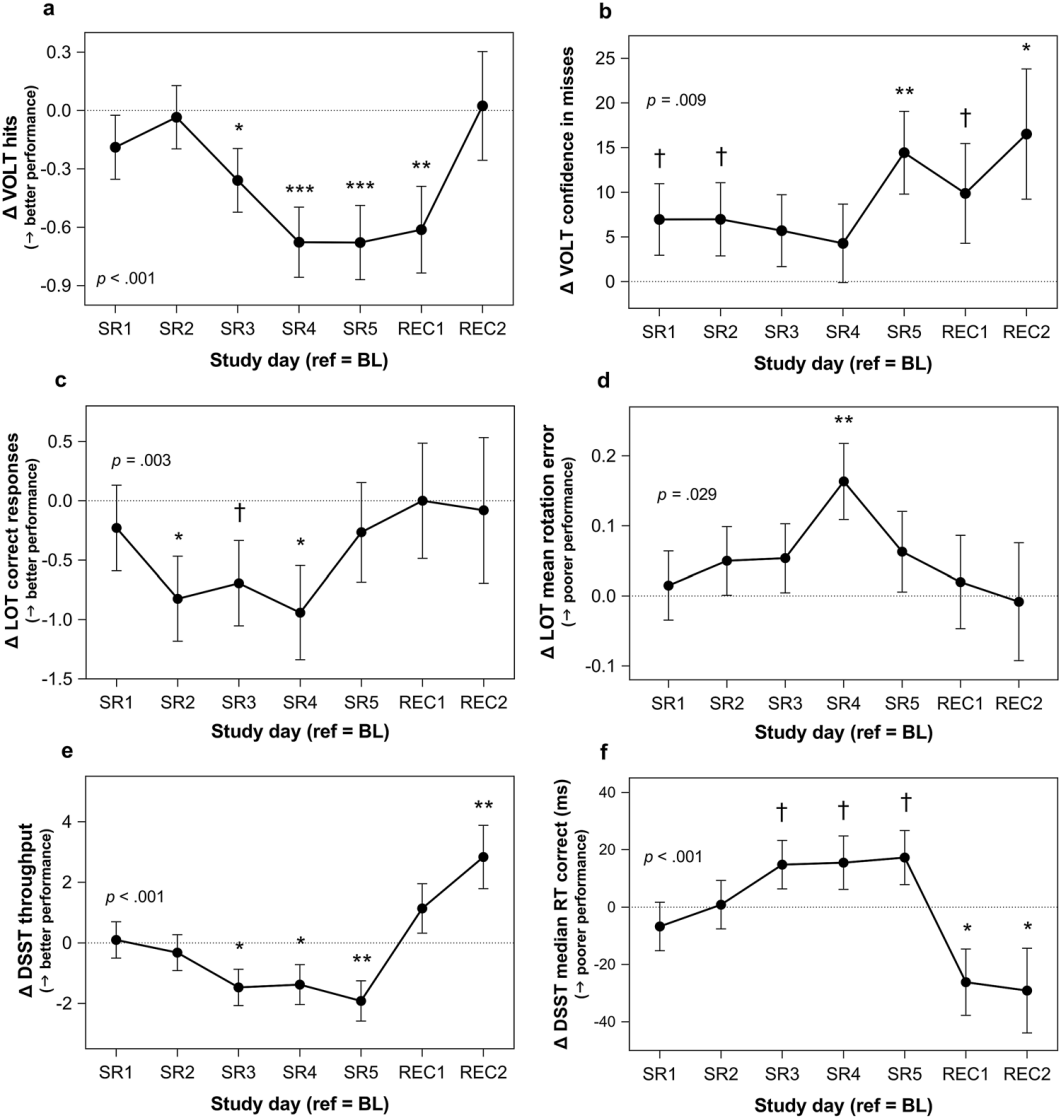
Working memory, visuospatial processing, and processing speed performance across sleep restriction (change from baseline). (**a**) Visual Object Learning Task (VOLT)^40^ hits are number of true positives (indicating “definitely yes” or “probably yes” in the recall phase when the image had been presented during the learning phase). (**b**) VOLT confidence in misses is calculated as the number of “definitely no” misses divided by the total number of misses (“definitely no”+ “probably no”), multiplied by 100. (**c**) Line Orientation Task (LOT)^48^ correct responses are the number of target lines (out of 24) correctly rotated to be parallel to the sample line. (**d**) LOT mean rotation error is the average rotation error from the correct line orientation (with 0 indicating a correct response, the target line exactly parallel to the sample line) for all 24 lines. (**e**) Digit Symbol Substitution Task (DSST)^49^ throughput is the number of correct responses/minute per test. (**f**) DSST median reaction time (RT) correct is the median RT for all correct responses per test (ms). Change from baseline (BL) values are plotted; horizontal dotted line indicates the baseline reference level (where change is 0). Comparisons to the BL reference point (the day succeeding the third BL night) during each sleep restriction (SR) and recovery (REC) day: ^†^*p* < .10, *p < .05, **p < .01, ***p < .001, two-tailed. Error bars depict standard error of the difference. Values are estimated from linear mixed modeling.

There were no significant interactions between vulnerability and *day* on any Visual Object Learning Task (VOLT) measure (all *p* > .300). Correct responses (*p* = .008, downward trajectory), hits (*p* < .001, downward trajectory; Fig. 2a), median RT correct (*p* < .001, downward trajectory), and confidence in incorrect responses (*p* = .005, upward trajectory) and in misses (*p* = .009, upward trajectory; Fig. 2b) changed significantly throughout the study at the mean of attentional vulnerability in linear trajectories. Confidence was computed as the percentage of “definitely [saw image]” or “definitely [did not see image]” responses versus “probably [saw image]” or “probably [did not see image]” responses per task, ranging from 0 (low confidence) to 100 (high confidence).Neither correct rejections nor confidence in false alarms (both *p* > .400) changed throughout the study at the mean of attentional vulnerability.

#### *Fractal 2-Back* (*working memory*)

**Figure 3:**
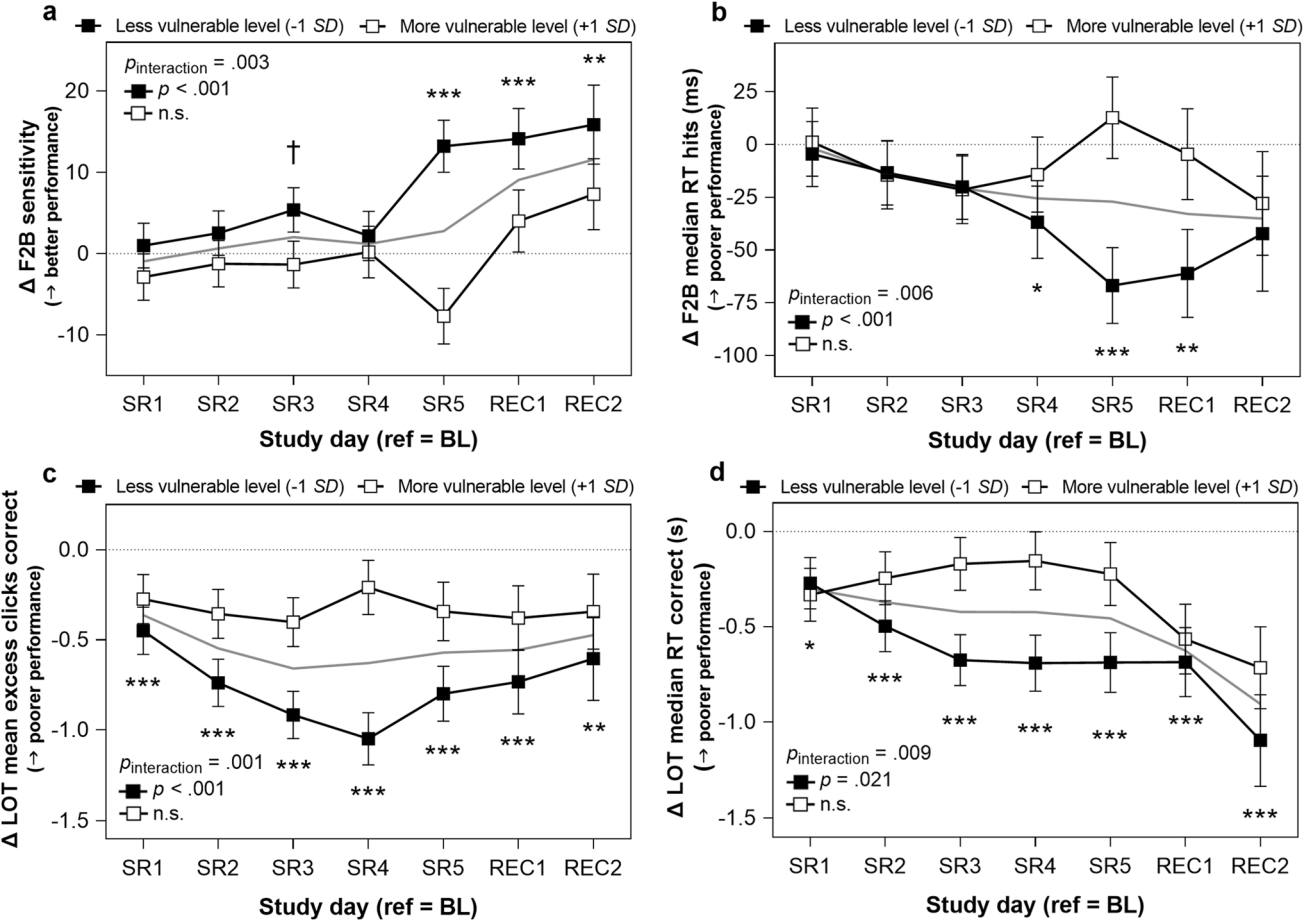
Working memory and visuospatial processing performance by vulnerability level per participant across sleep restriction (change from baseline). (**a**) Fractal 2-Back (F2B)^39^ sensitivity is calculated as 100 * (true positives/(true positives + false negatives)). False negatives occur when a participant has been presented with an image previously but fails to tap the screen. (**b**) F2B median reaction time (RT) for hits (ms). (**c**) Number of excess clicks for correct responses on the Line Orientation Task (LOT)^48^. (**d**) LOT median RT correct is the median RT for all correct responses per test (s). “Less vulnerable level” estimates were obtained by re-centering attentional vulnerability (i.e., difference in mean psychomotor vigilance task lapses on last sleep restriction day relative to the last baseline day for each participant) at 1 standard deviation (*SD*) below the mean. “More vulnerable level” estimates were obtained by re-centering attentional vulnerability at 1 *SD* above the mean (*N* = 15 in all analyses; refer to Statistical Analyses). See Fig. 6 for vulnerability level per participant. Change from baseline (BL) values are plotted; horizontal dotted line indicates the baseline reference level (where change is 0). Comparisons to the BL reference point (the day succeeding the third BL night) during each sleep restriction (SR) and recovery (REC) day separated by vulnerability level: ^†^*p* < .10, *p < .05, **p < .01, ***p < .001, two-tailed. Comparisons to baseline not completed at “more vulnerable level” for any measure due to lack of significant trajectory (see Table 1, “by vulnerability” column). Performance at the mean of attentional vulnerability is represented by the gray line. Error bars depict standard error of the difference. Values are estimated from linear mixed modeling.

There were significant interactions between vulnerability and *day* on Fractal 2-Back (F2B) accuracy (*p* = .006), sensitivity (true positives/images warranting a screen tap; *p* = .003; Fig. 3a), and median RT for hits (*p* = .006; Fig. 3b). All three measures improved significantly throughout the study at the less vulnerable level in linear trajectories (all *p* < .001). These less vulnerable trajectories contrast with unchanged accuracy, sensitivity, and median RT for hits at the more vulnerable level during the study (all *p* > .200). Vulnerability did not moderate the impact of sleep restriction on specificity (true negatives/images warranting no screen tap; *p* = .420). Specificity did not change across study days at the mean of vulnerability (*p* = .165).

#### *Line Orientation Task* (*visuospatial processing*)

There were significant interactions between vulnerability and *day*^2^ on the mean number of excess clicks for correct responses (*p* = .001; Fig. 3c) and median RT correct (*p* = .009; Fig. 3d) on the Line Orientation Task (LOT). Both mean number of excess clicks for correct responses (*p* < .001) and median RT correct (*p* = .021) exhibited U-shaped trajectories at the less vulnerable level, decreasing on all sleep restriction days and both recovery days compared to baseline. Neither mean number of excess clicks for correct responses nor median RT correct changed at the more vulnerable level (both *p* > .080). Vulnerability did not moderate the impact of sleep restriction on either correct responses or mean rotation error (ranging from 0, lowest to 30, highest; both *p* > .800). Both correct responses (*p* = .003, U-shape; Fig. 2c) and mean rotation error (*p* = .029, inverse U-shape; Fig. 2d) changed significantly across study days at the mean of vulnerability in quadratic trajectories.

#### *Digit Symbol Substitution Task* (*processing speed*)

Vulnerability did not moderate the impact of sleep restriction on DSST throughput (number correct per minute) or median RT for correct responses (both *p* > .100). Both throughput (U-shape; Fig. 2e) and median RT for correct responses (inverse U-shape; Fig. 2f) exhibited quadratic trajectories throughout the study (both *p* < .001).

## Discussion

The current study examined whether interindividual vulnerability to the attentional effects of sleep restriction, defined as the change in lapses of attention per participant from baseline sleep repletion to sleep restriction, moderated the impact of sleep restriction and subsequent recovery sleep on working memory, visuospatial processing, and processing speed. We demonstrated that comparatively less vulnerable participants exhibited practice improvements that were masked in more vulnerable participants; they improved accuracy overall, improved sensitivity, and exhibited faster RT for hits throughout the study on a task of working memory (the F2B). Furthermore, both mean excess clicks and median RT for correct responses improved on the LOT of visuospatial processing in comparatively less vulnerable participants throughout the study, but these measures did not change in more vulnerable participants. The performance improvements in comparatively less vulnerable participants were evident despite sleep restriction and persisted into the recovery period. These findings suggest that interindividual vulnerability to more attentional lapses during sleep restriction can explain differences in forgetting but not false memories on a difficult, fast-paced task of working memory, as well as greater improvement across the study (which included five days of sleep restriction) in speed and efficiency on a task of visuospatial processing. Other aspects of cognitive performance were impaired during sleep restriction regardless of attentional vulnerability, including decreased hits on the VOLT of working memory (persisting into the first recovery day), decreased correct responses and increased mean rotation error on the LOT of visuospatial processing, and decreased throughput and slower median RT for correct responses on the DSST of processing speed.

F2B performance improvement across the study (increased sensitivity and shorter median RT for hits) was evident at a lower level of attentional vulnerability but not evident at a higher level, supporting the existing evidence that attention plays an important role in recall of items stored in working memory. The time-based resource sharing (TBRS) framework posits that memory decay is prevented by continuous attentional focusing on the items to be remembered^37^. With regard to visual memory specifically, the same neurons in the frontal eye field that are active during presentation of a cued stimulus, indicating increased attention to that stimulus, are also active throughout the period between memory encoding and retrieval (i.e., the delay period) in monkeys^38^. The higher PVT lapses during sleep restriction and masked practice improvement on the F2B exhibited by more vulnerable participants in the current study may therefore reflect a global attentional deficit that simultaneously resulted in slower responses and in less capacity to attend to relevant stimuli during a working memory task. Future research may investigate whether participants more vulnerable to attentional impairment during sleep loss exhibit less attentional capacity on working memory tasks during encoding, during the delay period after encoding, during retrieval, or some combination.

Practice improvement was masked on the F2B of working memory in more vulnerable participants, but VOLT performance (hits) decreased during sleep restriction regardless of vulnerability level, potentially due to the more fast-paced nature of the F2B. During the F2B, participants need to tap the screen in a fixed window of 1.7 s when they see an image that had been shown two images back^39^. If a participant does not respond within this window, their sensitivity will decrease. This contrasts with the VOLT, in which participants are allowed unlimited time to choose a response^40^. It is possible that the mechanisms that slow responses on the PVT in certain participants (making them “vulnerable”) during sleep restriction are similar to those that reduce sensitivity on the F2B, which is a fast-paced task of working memory. This is supported by the fact that F2B median RT for hits showed a similar pattern to sensitivity, becoming faster in less but not more vulnerable participants throughout the study. Research has suggested “neuronal lapses” as a potential mechanism for neurobehavioral lapses on the PVT during total sleep deprivation^41^. Neuronal lapses occur when neuronal spiking in certain brain regions (e.g., the medial temporal lobe) in response to PVT stimuli are attenuated, delayed, and lengthened immediately before slow behavioral responses compared to fast responses. In contrast to the F2B, VOLT median RT for correct responses became faster regardless of vulnerability level, suggesting that speed in more vulnerable participants may improve despite sleep restriction and throughout recovery at a memory task which is less difficult. Interestingly, confidence in misses increased throughout the study regardless of vulnerability, suggesting that participants may have been unaware of their increased forgetting during sleep restriction and into the first recovery day.

The present study demonstrated that regardless of vulnerability level, participants made fewer VOLT hits during sleep restriction and into the first recovery day compared to baseline beginning on the third sleep restriction day. Because tasks of working memory are more complex than simple tasks of sustained vigilance^29^, participants may have engaged in compensatory mechanisms that temporarily sustained working memory performance at the beginning of the sleep restriction period. Such compensatory mechanisms may occur by increased functional connectivity between the dorsal and ventral default mode networks^42^ or increased prefrontal and thalamic activation^43^ during sleep deprivation. Studies of compensatory mechanisms for performance on complex tasks during sleep restriction specifically, and their time courses, are warranted. Furthermore, VOLT hits rebounded to baseline levels only after the second recovery night. Such findings suggest that working memory deficits may persist even after one night of 10-h recovery TIB.

Neither VOLT correct rejections nor F2B specificity changed across the study, regardless of vulnerability, which is inconsistent with some literature suggesting that sleep loss leads to false memories^44–46^. However, the paradigms employed in these studies involved long-term memory tasks, with encoding in the evening and retrieval in the morning. It is possible that the increased delay between encoding and retrieval compared to working memory tasks such as the VOLT and F2B allowed for formation of false memories during sleep loss in the previous studies. Future research is needed to examine whether attentionally vulnerable individuals are more susceptible to creation of false memories during sleep loss when the delay between encoding and retrieval is more prolonged, allowing for more memory consolidation processes.

On the LOT, a test of visuospatial processing, fewer clicks and faster median RT for correct responses indicate greater response efficiency; less time and fewer attempts are required for a participant to achieve the correct response. In vigilance tasks, a signal is presented until a response indicating signal detection is made, and the measure of efficiency is known as the hit rate^47^. Fewer lapses on the PVT, then, indicate the participant is detecting the signals in a relatively short period of time, analogous to achieving more hits. On the LOT, whether the lines are parallel may be considered the “signal.” The present findings of a stronger practice improvement in mean excess clicks and median RT for correct responses in less vulnerable participants suggest that the ability to detect the “signal” of a parallel line on the LOT may be impaired during sleep restriction in participants that also demonstrate fewer “hits” in a simple response time task, the PVT. The potential impairment in signal detection on the LOT in more vulnerable participants may have caused the masked practice effect in excess clicks and median RT for correct responses that remained unmasked in less vulnerable participants.

Regardless of vulnerability level, participants made fewer correct responses and exhibited more rotation error on the LOT of visuospatial processing and had lower throughput and slower median RT for correct responses on the DSST of processing speed during sleep restriction, with performance rebounding after one recovery night. This contrasts with the practice improvements in working memory on the F2B and in visuospatial efficiency on the LOT that were evident in less vulnerable participants. These contrasting findings may be due to the differences in the cognitive domain assessed by each performance metric^37^. The LOT is a task of visuospatial processing, and accuracy on the LOT (as indicated by more correct responses and lower mean rotation error) depends on attending to spatial cues^48^. Similarly, the DSST requires spatial attention, as the task activates multiple regions of the brain, including portions of the frontoparietal network^49^ that are involved in processing spatial cues^50^. Spatial attention may be an aspect of performance that deteriorates during sleep restriction, regardless of attentional vulnerability measured through lapses on the PVT, and does not necessarily depend on speed and efficiency.

The current study had some limitations. The sample size was somewhat small, and future studies would benefit from a larger number of participants. Only young males were recruited, limiting the generalizability of the present study. Future studies should examine whether these findings are applicable to females and/or older adults. Another limitation is that positive bias may be present when researchers test the significance of an effect (i.e., day) at specific points of the moderator (i.e., attentional vulnerability), as in the pick-a-point approach used in the current study^51^. All participants experienced the inpatient protocol in the same order: baseline, sleep restriction, and recovery. The order of these conditions was not randomized, resulting in the potential for order effects on cognitive performance. Though we excluded the first two baseline days to mitigate practice effects, performance improved past the last baseline day and throughout sleep restriction and recovery on some measures, such as VOLT median RT for correct responses. Therefore, our findings may underestimate the effects of sleep restriction on performance, particularly in more attentionally vulnerable individuals. Also, the present study did not manipulate attentional performance during sleep loss. Therefore, we cannot conclude from the current findings that reduced attentional resources *caused* poorer performance in certain individuals.

Our findings indicate that individuals with poorer vigilance after several days of sleep restriction are more susceptible to masked performance improvement on working memory sensitivity and visuospatial processing speed and efficiency. Impaired vigilance may therefore be an important between-persons marker of domain-specific performance. The findings from the current study bear implications for situations where unimpaired performance is vital for optimal functioning. For example, short sleep in both the workplace^52^ and academic settings^53–55^ is associated with impaired performance on tasks requiring working memory, which our findings suggest is particularly impaired in attentionally vulnerable individuals. Moreover, the results from the current study suggest one night of recovery sleep is insufficient to return working memory performance to baseline levels following sleep restriction across individuals. Impaired visuospatial processing resulting from insufficient sleep may be associated with dangerous traffic conditions. Studies of the effects of short sleep on operating motor vehicles demonstrate increased lane drifting in both simulated^3,56,57^ and real-life^57^ driving conditions. Increased incidence of lane-drifting during sleep loss may signify perceptual decrements in tracking of road lines while driving. Given that about 35% of American adults report obtaining fewer than the minimum recommended amount of seven hours of sleep on an average night^58^, millions of American adults may be susceptible to decreased performance resulting from insufficient sleep, particularly those more vulnerable to attentional impairment during sleep loss.

## Methods

### Participants

Eligible participants were male, 20–35 years of age, and in good physical and mental health, verified by comprehensive history and physical exam, laboratory tests, and interview with a clinical psychologist. Females were excluded due to the effects of the female menstrual cycle on circadian rhythms^59^, and those over 35 were excluded due to potential age-related changes in sleep, including decreased deep sleep and poorer sleep quality with advancing age^60^.

### Procedure

#### Recruitment and screening

The study protocol was approved by the Institutional Review Board of The Pennsylvania State University, and all procedures conformed to the principles established by the *Declaration of Helsinki*. All participants provided written informed consent for both screening and inpatient procedures and were compensated for participation in the study.

Participants were recruited through posted flyers, advertisements, and research websites. Interested individuals completed a secure online screening questionnaire (Qualtrics, Seattle, WA) and eligible individuals were contacted by study staff. Individuals were excluded for tobacco or drug use (confirmed by urine toxicology), prescription medication use, chronic medical disorders, hearing/vision impairment, neurological or sleep disorders, night or shift work within the previous three years, or travel across > 2 time zones within the previous 3 months. Those who successfully completed screening visits and met study criteria were invited to participate in the 11-day inpatient protocol.

#### Pre-study monitoring

Pre-study sleep monitoring was conducted to ensure all participants maintained the sleep–wake behaviors imposed by the study protocol prior to admission into the study . Participants were instructed to spend 10 h in bed from 22:00 to 8:00 (± 1 h) for at least one week prior to the inpatient stay, verified by wrist actigraphy, time-stamped call-ins at bedtime and wake time, and sleep/wake logs. During this period, participants were instructed to discontinue alcoholic beverages, caffeine, and over-the-counter medications.

Actigraphy data from the pre-study monitoring were downloaded using Philips Actiware software (versions 6.0.4. and 6.0.9.). At least two independent scorers (blinded to each other) determined “day” cut-point times, validity of days, and set sleep intervals using a previously validated algorithm^61^, without using information from the sleep/wake log. First, scorers reviewed recordings for participant compliance, ensuring the device was worn daily, except for permitted periods of removal when the watch could be damaged, such as during contact sports. The scorers adjudicated each recording for inter-rater agreement by verifying number of valid days, cut point, number of sleep intervals, and differences greater than 15 min in duration and wake after sleep onset for each sleep interval. Specifically, trained scorers determined sleep intervals using a decrease in activity levels and the aid of light levels for sleep onset and sleep offset^62^, and a nighttime sleep interval was split into two intervals (main sleep and nap) if there was an awakening ≥ 1 h during this interval. A sleep actigraphy day was determined invalid and no sleep interval was set if there were ≥ 4 total hours of off-wrist time, except the first and last day (device should have been worn at least 2 h before sleep onset on the first day), constant false activity due to battery failure, data unable to be recovered, or an off-wrist period of ≥ 60 min within 10 min of the scored beginning or end of the main sleep period for that day. Measures of interest calculated by actigraphy included sleep onset, sleep midpoint, sleep offset, total sleep time (TST), and sleep maintenance efficiency for the main nighttime sleep interval.

#### Inpatient study

The inpatient study took place across 10 nights (11 days). Participants were admitted to the Clinical Research Center of the Pennsylvania State University at approximately 11:00 on admission day to a private, windowless room under constant (artificial) light levels (< 100 lx in the angle of gaze during wake; complete dark at 0 lx during scheduled sleep) and temperature conditions (20–22 °C). Participants remained in the room until approximately 16:00 on the last inpatient day. The private room contained a single bed, a desk for administration of cognitive batteries, and a bathroom with a shower. Participants were not permitted to nap, sit, or recline in bed during scheduled wake times and were monitored by research assistants to confirm adherence. Light-emitting personal devices such as mobile phones and laptop computers were removed 2 h before scheduled bedtime and were returned at least 2 h after scheduled wake time to limit exposure to the alerting effects of blue light near the sleep episode^63^.

**Figure 4:**
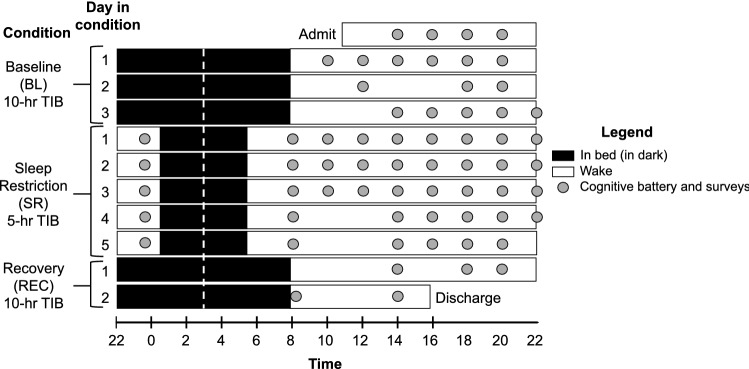
Depiction of 11-Day (10-Night) Inpatient Study Protocol*.* Black bars represent time in bed (TIB) in dark conditions; white bars represent supervised wake. The first three sleep periods were 10-h TIB baseline (BL) from 22:00 to 08:00. Following BL, participants had 5-h TIB sleep restriction (SR) for 5 nights with TIB from 00:30 to 05:30. Afterward, participants had two 10-h TIB recovery (REC) nights, again from 22:00 to 08:00. All sleep periods were centered at 03:00 to minimize circadian misalignment (white dotted line). Approximately every two hours during wake, participants completed the 20-min cognitive battery (gray dots) including the 10-min psychomotor vigilance task (PVT).

The protocol consisted of admission day, three baseline nights with 10 h TIB, five sleep restriction nights with 5 h TIB, and then two recovery nights with 10 h TIB. In both baseline and recovery conditions, bedtime was 22:00 and wake time was 8:00. During sleep restriction, bedtime was 0:30 and wake time was 5:30. Inpatient sleep was measured using PSG (Nihon Kohden, Irvine, CA) including electroencephalographic (EEG) electrode placement consistent with American Academy of Sleep Medicine (AASM) recommendations (Berry et al., 2015; Berry et al., 2017). Sleep was staged in 30-s epochs by a registered polysomnographic technologist according to AASM standards^64,65^. Nights with ≥ 5.5% unscorable data within the sleep opportunity interval (lights-off to lights-on, recorded by study staff) due to disconnection or artifacts were excluded from analyses. During the study, participants were permitted to engage in activities such as reading, completing puzzles, light stretching, and browsing the internet, provided no study procedures were scheduled at that time. See Fig. 4 for a depiction of the 11-day protocol.

### Measures

#### Cognitive tasks

**Figure 5:**
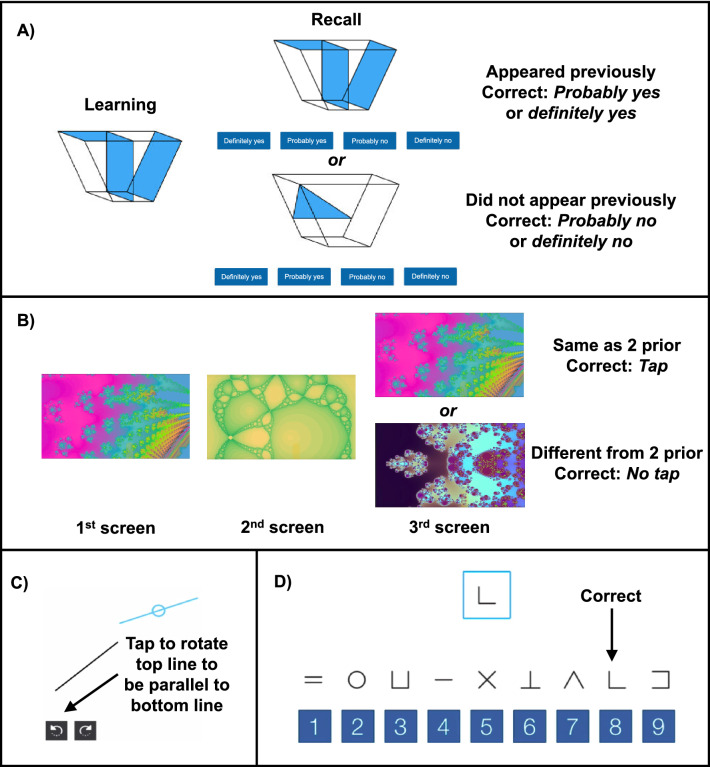
Depiction of The Cognitive Tasks During the Inpatient Study. (**A**) During the learning phase of the Visual Object Learning Task (VOLT)^40^ of working memory, participants are presented with ten objects displayed for five seconds each. During the succeeding recall phase of the VOLT, participants are presented with ten novel objects and ten seen objects and indicate whether they had seen the object. Correct responses are indicating “probably yes” or “definitely yes” when the object had been presented (hits), *or* indicating “probably no” or “definitely no” when the object had not been presented (correct rejections)*.* (**B**) In the Fractal 2-Back (F2B)^39^ of working memory, participants are presented with sequential images for 1.7 s each and asked to tap the screen when an image appears that has appeared two images prior. Correct responses are tapping the screen when an image had been presented previously (hits, which increase sensitivity)*, or* withholding a response when an image had not been presented previously (correct rejections, which increase specificity). (**C**) In the Line Orientation Task (LOT)^48^ of visuospatial processing, participants are asked to tap the bottom arrows to incrementally rotate the target line to be parallel to the target black line. (**D**) In the Digit Symbol Substitution Task (DSST)^49^ of processing speed, participants are asked to tap the number which corresponds to the symbol displayed at the top of the screen. In all tasks, participants are asked to respond as accurately and as quickly as possible. Images are copyright Joggle Research and reproduced with permission.

Approximately every two hours during wake time, participants completed the validated Joggle Research cognitive battery^28^, which lasted about 20 min, seated at the same desk on a touchscreen tablet, angled at approximately 120° and 32 cm from the desk edge. All eight Joggle tests were administered in sequence. For all tests, participants were instructed to respond as accurately and as quickly as possible. Participants were continuously monitored to ensure adherence and to document test validity. A test was deemed invalid and removed from analyses in the rare event of an external distraction (e.g., unexpected interruption) or technical error (e.g., program crashed). See Fig. 5 for depiction of the tests of working memory, the VOLT^40^ and F2B^39^; visuospatial processing, the LOT^48^; and processing speed, the DSST^49^. Each cognitive outcome was chosen a priori for analysis.

#### PVT and attentional vulnerability calculation

**Figure 6:**
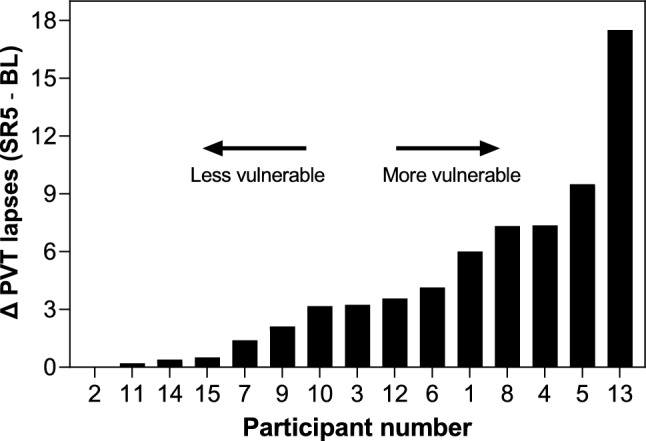
Computation of Attentional Vulnerability Per Participant. The mean difference in the number of lapses per 10-min psychomotor vigilance task (PVT) during the last sleep restriction day (SR5) versus the last baseline day (BL) are depicted in ascending order, indicating the vulnerability per participant. Vulnerability per participant was used as a continuous moderator in analyses of cognitive variables.

During the validated 10-min PVT^66,67^, the participant fixated on an empty red square and was instructed to tap the screen as quickly as possible when a counter appeared (counting RT displayed on the screen in ms), while avoiding tapping prematurely. Upon tapping, the counter stopped, displaying the achieved RT in ms. Random interstimulus intervals ranged from 2 to 10 s. *Lapses* indicate errors of omission and occur when a participant has an RT ≥ 500 ms. Attentional vulnerability to the effects of sleep loss per participant was calculated as the difference between the mean number of lapses (within-person) a given participant made on the last day of sleep restriction versus the mean on the last day of baseline per 10-min PVT (see Fig. 6). We chose to keep the moderator continuous instead of an arbitrary median split, which would place participants with widely different attentional vulnerability levels in the same group.

##### VOLT

The VOLT^40^ assesses visual working memory using images of differently shaded geometric shapes. During the learning phase of the VOLT, the participant was presented with ten consecutive shapes for five seconds each and was instructed to do their best to remember each entire shape. During the recall phase which immediately followed, the participant was presented with twenty shapes: ten of which were presented during the learning phase, and ten novel shapes. The participant indicated whether the shape was presented in the learning phase by tapping one of four buttons: “Definitely yes,” “probably yes,” “definitely no,” or “probably no.” Outcomes from the VOLT were assessed from responses during the recall phase. *Correct responses* (range: 0–20) include the sum of *hits* and *correct rejections* (each range: 0–10)*.* When the shape had been presented during the learning phase, *hits* indicate the number of responses during the recall phase wherein the participant selected “definitely yes” or “probably yes.” When the shape had *not* been presented during the learning phase, *correct rejections* indicate the number of responses wherein the participant selected “definitely no” or “probably no.” *Median RT correct* is the median response latency in ms for correct responses only. We also assessed *confidence in incorrect responses* (incorrect responses = 20 – *correct responses*), *confidence in misses* (misses = 10 − *hits*), and *confidence in false alarms* (false alarms = 10 − *correct rejections*). Confidence was computed as the number of “definitely” responses over the total number of responses of that type, multiplied by 100. For example, confidence in misses was calculated as the number of “definitely no” misses divided by the total number of misses (“definitely no” + “probably no”), multiplied by 100. Values may range from 0 to 100, with values closer to 100 indicating greater response confidence, and values closer to 0 indicating less confidence.

##### F2B

The F2B^39^ assesses visual working memory using images of abstract patterns. Participants were instructed to tap the screen when an image appeared that had appeared two images back (i.e., “2-back”) but withhold a response otherwise, allowing a screen timeout. Images disappeared from the screen upon tap or after 1.7 s with no tap. *Accuracy* is the number of correct responses (hits or correct rejections) divided by the total number of presented stimuli and multiplied by 100. *Sensitivity* is the total number of hits divided by the total number of images warranting a screen tap (i.e., divided by the sum of hits and misses), multiplied by 100. *Specificity* is the number of correct rejections divided by the total number of images warranting no response (i.e., divided by the sum of correct rejections and false alarms), multiplied by 100. Accuracy, sensitivity, and specificity may range from 0–100. *Median RT for hits* was also examined in ms (RT for correct rejections was always 1.7 s and was therefore not examined).

##### LOT

The LOT^48^ assesses visuospatial processing. During the LOT, the participant was presented with two lines, a sample black line and a blue target line. The participant incrementally rotated the target line to be parallel to the sample line by tapping the right or left arrow which rotated the target line clockwise or counterclockwise, respectively. *Correct responses* indicate the number of trials (out of 24 for each administration) the participant correctly matched the target line’s orientation to the sample line (range: 0–24). *Mean rotation error* indicates the mean number of clicks the participant deviated from the correct line orientation in all 24 trials and ranged from 0 (no rotation error) to 30 (perpendicular line; highest rotation error). *Mean excess clicks correct* indicate the mean number of excess clicks the participant made for correct responses. *Median RT correct* was also examined in seconds.

##### DSST

The DSST^49^ assesses processing speed during a visual search. Participants were presented with a symbol at the top of the screen and tapped the number corresponding to that same symbol at the bottom of the screen. *Throughput* is the number of correct responses per minute of the task. *Median RT correct* was also examined in ms.

### Statistical analyses

Analyses were conducted in SAS 9.4 (SAS Institute Inc., Cary, NC). To determine the practice improvement across baseline days, we examined the trajectory of performance for each cognitive outcome across the three baseline days in linear mixed models. Performance significantly improved (all *p* < .04) across the first three baseline days on four (VOLT median RT for correct responses, LOT excess clicks for correct responses and median RT for correct responses, and DSST throughput) out of all 15 cognitive outcomes considered (response confidence on the VOLT not included). In all four cases, performance was best on the fourth baseline day (see Supplementary Table S5 for descriptives and comparisons of performance across baseline days). Due to practice improvement across multiple administrations of the cognitive tasks^28^, we used the third baseline day (following 10-h TIB sleep period 3) as the reference point. Unless mentioned otherwise, all analyses were mixed models with a random intercept for participants, maximum likelihood estimation, and autoregressive covariance structure, AR(1). The moderator had skew <|3| and kurtosis <|10|, and all outcomes met these normality standards^68^ for each day of the study. Alpha < .05 (two-sided) was deemed statistically significant.

The changes during the pre-study monitoring in sleep onset, midpoint, offset, TST, and sleep maintenance efficiency among habitual, pre-study, and in-lab baseline conditions as measured with actigraphy were analyzed with mixed models with a fixed effect of condition (night 1 of baseline, the habituation night, was excluded due to the first night effect)^69^. The changes during the inpatient study in TST among baseline, sleep restriction, and recovery conditions as measured with PSG were also analyzed with mixed models with a fixed effect of condition (night 1, the habituation night, was excluded due to the first night effect)^69^. In both sets of analyses, a significant effect of condition was followed with analyses of each pairwise comparison, corrected with Tukey’s Honestly Significant Difference (HSD) test.

#### Interactions between vulnerability and sleep restriction on cognitive performance

The statistical analyses were designed to test the hypothesis that attentional vulnerability per participant (a between-person variable measuring interindividual differences) moderated performance across the study within each person^70^. The metrics from each administration of a cognitive task were entered into analyses. Estimated differences from baseline for each sleep restriction and recovery day using the effect of “day” (the reference was the last baseline day) were generated from statistical models for graphing purposes. For each outcome, the best fitting out of three models was selected as the final model, determined by change in − 2 log likelihood (− 2LL). As − 2LL follows a chi-square distribution, the model with additional terms was selected as a better fit if the difference between the − 2LL of adjacent models exceeded the critical chi-square value. The first model included study day (the last baseline day, five sleep restriction days, and two recovery days) and its interaction with a participant's vulnerability level (day*vulnerability); the second model examined the potential (polynomial) quadratic trajectory (*day*^2^*vulnerability), and the third model added *time of day of task administration.* We chose to model these measures as a function of *day* or *day*^2^ rather than *condition* because the effects of sleep loss on performance may progressively accumulate over several days^4,7,10^.

For interpretation of an interaction with a continuous moderator, values of interest for the moderator are chosen in the “pick-a-point” approach^51,71,72^. If there are no meaningful values a priori, often 1 *SD* below and 1 *SD* above the mean are selected as “low” and “high” values of the moderator, respectively. For significant interactions, as the moderator was continuous, we investigated the effect of *day* or *day*^2^ on the outcome at the low (“less vulnerable level”) and high (“more vulnerable level”) values of the moderator (attentional vulnerability per participant). To obtain these effects, attentional vulnerability was centered at 1 *SD* below and at 1 *SD* above the mean. The analyses were re-run with the re-centered attentional vulnerability^51^, again with all 15 participants in the model. The effect of *day* or *day*^2^ at 1 *SD* above or below the mean of attentional vulnerability is represented by the effect in a model with attentional vulnerability centered at 1 *SD* above or below its mean (rather than a model with “raw” attentional vulnerability). If the interaction was not significant, the analysis was re-run with attentional vulnerability centered around its mean, with the effect of *day* or *day*^2^ indicating the effect at the mean of attentional vulnerability. If the effect of *day* or *day*^2^ was significant in any analysis, subsequent analyses compared performance on each sleep restriction and recovery day to the baseline reference day. Measures of effect size for the predictor of interest in each model (R^2^) were included in tables and computed as (*variance in model without predictor – variance in model with predictor*)*/variance in model without predictor*^73^.

To investigate whether the results remained after accounting for differences in sleep rebound among individuals, we re-conducted the analyses adjusted for the change in sleep efficiency from baseline to recovery (calculated as *sleep efficiency on first recovery night* − *sleep efficiency on last baseline night*) per participant. These analyses yielded the same results as primary analyses and are therefore not included. We additionally extracted cognitive factors using PFA for dimension reduction and re-conducted the main analyses with the cognitive factors as outcomes (see Supplementary Information for analytical details and results). Supplementary Table S6 displays factor loadings for variables extracted through PFA.

## Supplementary Information

Supplementary Information.

## Data Availability

The data that support the findings of this study are available on request, due to privacy or other restrictions, through co-author Dr. Anne-Marie Chang (amchang@psu.edu).
