# Supplementary material for: Interindividual differences in attentional vulnerability moderate cognitive performance during sleep restriction and subsequent recovery in healthy young men
Source: Sci Rep. 2021 Sep 27;11:19147. doi: 10.1038/s41598-021-95884-w (PMC8476607; doi:10.1038/s41598-021-95884-w)
Supplement: Supplementary file 1 — Supplementary Information. [file 41598_2021_95884_MOESM1_ESM.pdf]

SUPPLEMENTARY INFORMATION

Title:

Interindividual Differences in Attentional Vulnerability Moderate Cognitive Performance During Sleep Restriction and Subsequent Recovery in Healthy Young Men

Authors and author affiliations:

Gina Marie Mathew*[1], Stephen M. Strayer[1], Kelly M. Ness[1,2], Margeaux M. Schade[1], Nicole G. Nahmod[1,3], Orfeu M. Buxton[1], & Anne-Marie Chang[1,4]

[1]Department of Biobehavioral Health, College of Health and Human Development, Pennsylvania State University, University Park, PA, USA

[2]Current address: Department of Medicine, Division of Metabolism, Endocrinology, and Nutrition, University of Washington, Seattle WA, USA

[3]Current address: Department of Medical Science, Arcadia University, College of Health Sciences, Glenside, PA, USA

[4]College of Nursing, Pennsylvania State University, University Park, PA, USA

***Corresponding author**:

Gina Marie Mathew

219 Biobehavioral Health Bldg

University Park, PA 16802

gina.marie.mathew@gmail.com

Results

Interactions between study day and attentional vulnerability on cognitive factors

See Supplementary Table S3 for interactions between attentional vulnerability (defined as change in mean psychomotor vigilance task, PVT [1, 2], lapses between last baseline day and last sleep restriction day) and *day* or day^2 (linear or quadratic trajectory for study day, depending on best fit; linear mixed model analyses included last baseline day, five sleep restriction days, and two recovery days) on factors extracted through principal factor analysis (PFA) and Supplementary Table S4 for comparisons of performance on each sleep restriction and recovery day to the baseline reference day (succeeding the third 10-hour time in bed sleep period).

Speed and efficiency

There was a significant interaction between *day* and attentional vulnerability on the speed and efficiency factor [which included 6 variables: Visual Object Learning Task (VOLT) [3], Line Orientation Task (LOT) [4], and Digit Symbol Substitution Task (DSST) [5] median reaction time (RT) correct, Fractal 2-Back (F2B) [6] median RT for hits, LOT excess clicks correct, and DSST throughput] (p = .003). Speed and efficiency improved across the study (p < .001) at the less vulnerable level (i.e., at 1 *SD* below the mean of vulnerability) but not at the more vulnerable level (i.e., at 1 *SD* above the mean of vulnerability) (p = .058).

Recall

There was a significant interaction between *day* and attentional vulnerability on the recall factor (which included VOLT hits and F2B sensitivity) (p = .009). Recall improved across the study at the less vulnerable level (p = .016) but not at the more vulnerable level (p = .171).

Visuospatial accuracy

There was no interaction between *day*^{2} and attentional vulnerability on the visuospatial accuracy factor (which included LOT correct responses and mean rotation error) (p = .808), which exhibited a U-shape quadratic trajectory across the study (p = .002).

Methods

Statistical analyses

Factor extraction

We conducted PFA for dimension reduction across a subset of the cognitive variables of interest. Certain variables were excluded due to overlap with other cognitive variables of interest (PVT lapses; VOLT correct responses; and F2B accuracy) or no apparent direction for "better" or "poorer" performance (VOLT confidence in incorrect responses, in misses, and in false alarms). The 12 variables included in the PFA were VOLT hits, correct rejections, and median RT correct; F2B sensitivity, specificity, and median RT for hits; LOT correct responses, mean rotation error, excess clicks for correct responses, and median RT correct; and DSST throughput and median RT correct. Given the multi-level structure of the data (nested within each participant), we first disaggregated the within-person (level-1) and between-person (level-2) variance for each of these variables. Within-person variables were centered around the person mean, such that positive values indicated that value was higher than the person's own average during the entire study. Between-person variables were calculated as the mean per person, such that positive values indicated that person's average was higher than others' averages in the sample. Due to the small sample size for between-person variance (N = 15), PFA was only conducted on the within-person variance [7].

The SAS command PROC FACTOR was used to conduct PFA (method=principal) with oblique rotation (rotate=promax) [8]. Both VOLT correct rejections and F2B specificity had low

final communality estimates (the proportion of variance of the variable accounted for by the common factors; each less than 1% of the total communality), indicating they did not load heavily on at least one of the retained factors [8] and were removed from the PFA. The scree plot for the PFA of the remaining 10 variables depicted a point of inflexion of four, indicating three factors should be retained [8]. Factor loadings are in Supplementary Table S6. The first factor, named "speed and efficiency," included all four tasks' median RT variable, LOT mean excess clicks correct, and DSST throughput. The second factor, named "recall," included VOLT hits and F2B sensitivity. The third factor, named "visuospatial accuracy," included LOT correct responses and mean rotation error. For ease of interpretation, each extracted factor was subsequently rescaled from 0 (worst performance) to 100 (best performance). Analyses examined whether attentional vulnerability (see section "PVT and attentional vulnerability calculation" in main text) moderated the effect of *day* (linear trajectory across the study) or day^2 (quadratic trajectory), depending on best model fit, on each extracted factor. See main text, section "Interactions between vulnerability and sleep restriction on cognitive performance," for detailed description of analyses.

References

1. Basner, M. & Dinges, D. F. Maximizing sensitivity of the psychomotor vigilance test (PVT) to sleep loss. *Sleep* **34**, 581–591 (2011).
2. Dinges, D. F. & Powell, J. W. Microcomputer analyses of performance on a portable, simple visual RT task during sustained operations. *Behav. Res. Methods, Instruments, Comput.* **17**, 652–655 (1985).
3. Glahn, D. C., Gur, R. C., Ragland, J. D., Censits, D. M. & Gur, R. E. Reliability, performance characteristics, construct validity, and an initial clinical application of a Visual Object Learning Test (VOLT). *Neuropsychology* **11**, 602–612 (1997).
4. Moore, T. M. *et al.* Development of an abbreviated form of the Penn Line Orientation Test using large samples and computerized adaptive test simulation. *Psychol. Assess.* **27**, 955–964 (2015).
5. Usui, N. *et al.* Cortical areas related to performance of WAIS Digit Symbol Test: A functional imaging study. *Neurosci. Lett.* **463**, 1–5 (2009).
6. Ragland, J. D. *et al.* Working memory for complex figures: An fMRI comparison of letter and fractal n-back tasks. *Neuropsychology* **16**, 370–379 (2002).
7. Reise, S. P., Ventura, J., Nuechterlein, K. H. & Kim, K. H. An illustration of multilevel factor analysis. *J. Pers. Assess.* **84**, 126–136 (2005).
8. O'Rourke, N. & Hatcher, H. *A step-by-step approach to using SAS® for factor analysis and structural equation modeling*. (SAS Institute, 2013).

Tables

Table S1. Sleep timing, total sleep time, and sleep efficiency among habitual, pre-study monitoring, and in-lab baseline nights, measured with actigraphy.

	Mean (*SEM*) by Condition			$F(2,270)$	Pairwise Comparisons: Difference[a] (*SEM*)			Significant Differences
	H	P	BL		P vs. H	BL vs. P	BL vs. H	
Onset[b]	1:13 (0:16)	23:50 (0:17)	22:25 (0:21)	62.77***	−1.39*** (.18)	−1.42*** (.29)	−2.81*** (.28)	H > P > BL
Midpoint[b]	5:18 (0:15)	4:21 (0:16)	3:12 (0:19)	44.49***	−.96*** (.16)	−1.15*** (.25)	−2.11*** (.24)	H > P > BL
Offset[b]	9:24 (0:15)	8:52 (0:16)	7:59 (0:21)	13.88***	−.53* (.18)	−.89** (.29)	−1.42*** (.28)	H > P > BL
TST (hrs)	8.20 (.12)	9.04 (.15)	9.57 (.26)	17.94***	.84*** (.18)	.53 (.29)	1.37*** (.28)	H < P, BL
SME (%)	88.39 (1.20)	87.02 (1.22)	89.94 (1.35)	7.65***	−1.38* (.51)	2.93** (.81)	1.55 (.78)	H, BL > P

Notes. The mean number of habitual (H) nights was 9.6 (range: 3-12); the mean number of pre-study (P) nights was 6.6 (range: 5-7). The in-lab baseline (BL) condition was always 3 nights, but the first night was excluded from actigraphy analyses.

[a]*p* was corrected with Tukey's Honestly Significant Difference (HSD) test.

[b]Variable was centered around midnight in analyses (0:00).

BL, baseline nights 2-3 during in-lab study period; H, habitual nights; P, pre-study monitoring nights; SME, sleep maintenance efficiency; TST, total sleep time.

$^{*}p < .05$, $^{**}p < .01$, $^{***}p < .001$, two-tailed.

Table S2. Comparisons of PVT, VOLT, F2B, LOT, and DSST performance during sleep restriction (SR) and recovery (REC) days to baseline (BL).

Outcome	Level	BL[a] (ref)	SR: Difference (*SEM*) 1	SR: Difference (*SEM*) 2	SR: Difference (*SEM*) 3	SR: Difference (*SEM*) 4	SR: Difference (*SEM*) 5	REC: Difference (*SEM*) 1	REC: Difference (*SEM*) 2
**PVT**									
Lapses§	*MV*	10.35 (2.39)	**2.73 (1.09)**	**4.61 (1.09)**	**7.12 (1.09)**	**6.96 (1.23)**	**8.77 (1.29)**	**4.05 (1.43)**	.53 (1.65)
**VOLT**									
Correct responses	*Mean*	17.60 (.54)	−.20 (.21)	−.07 (.21)	**−.43 (.21)**	**−.71 (.23)**	**−.57 (.24)**	***−.53 (.28)***	.14 (.36)
Hits	*Mean*	8.56 (.37)	−.19 (.16)	−.03 (.16)	**−.36 (.16)**	**−.68 (.18)**	**−.68 (.19)**	**−.61 (.22)**	.02 (.28)
Median RT, correct (ms)	*Mean*	951.74 (39.89)	**−46.11 (17.50)**	**−66.74 (17.33)**	**−86.39 (17.41)**	**−56.24 (19.28)**	**−102.23 (20.32)**	**−125.32 (23.75)**	**−115.74 (29.78)**
Response confidence[b]									
Incorrect responses	*Mean*	69.31 (8.03)	4.11 (3.32)	4.89 (3.33)	**7.33 (3.31)**	3.21 (3.59)	**10.06 (3.88)**	***8.92 (4.64)***	**14.18 (6.10)**
Misses	*Mean*	71.47 (7.58)	***6.95 (4.01)***	***6.97 (4.10)***	5.70 (4.03)	4.27 (4.40)	**14.42 (4.63)**	***9.87 (5.59)***	**16.51 (7.28)**
**F2B**									
Accuracy[c]	*LV*	89.92 (1.58)	.56 (.88)	1.10 (.87)	**2.08 (.87)**	−.04 (.97)	**3.51 (1.02)**	**3.81 (1.18)**	**3.55 (1.55)**
Sensitivity[c]	*LV*	70.78 (4.49)	.99 (2.75)	2.53 (2.73)	***5.39 (2.75)***	2.17 (3.05)	**13.22 (3.21)**	**14.12 (3.72)**	**15.87 (4.86)**
Median RT, hits (ms)	*LV*	541.68 (33.39)	−4.56 (15.47)	−13.37 (15.36)	−20.06 (15.44)	**−36.82 (17.11)**	**−66.80 (18.02)**	**−61.15 (20.90)**	−42.26 (27.31)
**LOT**									
Correct responses	*Mean*	13.96 (.58)	−.23 (.36)	**−.82 (.36)**	***−.69 (.36)***	**−.94 (.40)**	−.26 (.42)	.00 (.49)	−.08 (.61)
Rotation error (*M*)[d]	*Mean*	.47 (.05)	.01 (.05)	.05 (.05)	.05 (.05)	**.16 (.05)**	.06 (.06)	.02 (.07)	−.01 (.08)
Excess clicks, correct (*M*)	*MV*	2.33 (.28)	**−.45 (.13)**	**−.74 (.13)**	**−.92 (.13)**	**−1.05 (.15)**	**−.80 (.15)**	**−.73 (.18)**	**−.60 (.23)**
Median RT, correct (s)§	*LV*	4.92 (.23)	**−.27 (.13)**	**−.50 (.13)**	**−.67 (.13)**	**−.69 (.15)**	**−.69 (.16)**	**−.69 (.18)**	**−1.10 (.24)**
**DSST**									
Throughput[e]§	*Mean*	55.57 (1.76)	.10 (.60)	−.32 (.60)	**−1.47 (.60)**	**−1.38 (.66)**	**−1.92 (.67)**	1.14 (.82)	**2.84 (1.04)**
Median RT, correct (ms)§	*Mean*	850.18 (28.82)	−6.77 (8.49)	.86 (8.46)	***14.81 (8.47)***	***15.48 (9.32)***	***17.28 (9.42)***	**−26.17 (11.58)**	**−29.10 (14.79)**

Notes: Vulnerability calculated as difference between within-person mean of psychomotor vigilance task (PVT) lapses on last sleep restriction day versus last baseline day, per person (see Fig. 6 for vulnerability level per participant). For significant *day* or *day*^{2}**attentional vulnerability* interactions, comparisons to baseline reference point (BL; day succeeding third BL night) were made at lower vulnerability (LV, less vulnerable) and at higher vulnerability (MV, more vulnerable) levels. LV estimates were obtained by re-centering attentional vulnerability at 1 standard deviation (*SD*) below the mean. MV estimates were obtained by re-centering attentional vulnerability at 1 *SD* above the mean ($N = 15$ in all analyses; refer to Statistical Analyses). For non-significant interactions, comparisons to BL are made at the mean of vulnerability. Comparisons to BL only made for outcomes demonstrating significant trajectories across study (see Table 1, "By Vulnerability").

BL, baseline; DSST, DSST, Digit Symbol Substitution Task (processing speed) [5]; F2B, Fractal 2-Back (working memory) [6]; LOT, Line Orientation Task (visuospatial processing) [4]; LV, more vulnerable; *M*, mean; MV, more vulnerable; PVT, psychomotor vigilance task (attention) [1, 2]; REC, recovery; RT, reaction time; s, seconds; *SEM*, standard error of the mean; SR, sleep restriction; VOLT, Visual Object Learning Task (working memory) [3].

[a]Last baseline day (succeeding third baseline night).

[b]Ranges from 0 (minimally confident) to 100 (maximally confident).

[c]Ranges from 0 (minimally accurate, sensitive, or specific) to 100 (maximally accurate, sensitive, or specific).

[d]Ranges from 0 (no rotation error) to 30 (perpendicular line; highest rotation error).

[e]Number correct per minute.

§Model includes *time of day*.

Bold values indicate SR or REC day was significantly different from BL ($p < .05$). ***Bold italic*** values indicate SR or REC day was marginally different from BL ($p < .10$).

Table S3. Interactions between day and attentional vulnerability on cognitive factor performance.

Outcome	Predictor	Interaction		By Vulnerability		
		b (SEM)	ΔR^2	Level	*b (SEM)*	ΔR^2
Speed and efficiency[a§]	Day	−.056** (.019)	.013	*LV*	.75*** (.12)	.052
				MV	.23 (.12)	.005
Recall[b]	Day	−.121** (.046)	.010	*LV*	.72* (.30)	.008
				MV	−.41 (.30)	.003
Visuospatial accuracy[c]	Day^2	.004 (.016)	< .001	*Mean*	.22** (.07)	.014

Notes: All factors scaled from 0 (worst performance) to 100 (best performance). Vulnerability calculated as difference between within-person mean of psychomotor vigilance task (PVT) lapses on last sleep restriction day versus last baseline day, per person (see Fig. 6). For significant *day* or *day^2*vulnerability* interactions, the effect of *day* or *day^2* at lower vulnerability (LV, less vulnerable) and at higher vulnerability (MV, more vulnerable) levels is shown. LV estimates were obtained by re-centering attentional vulnerability at 1 standard deviation (*SD*) below the mean. MV estimates were obtained by re-centering attentional vulnerability at 1 *SD* above the mean (*N* = 15 in all analyses; refer to Statistical Analyses). For non-significant interactions, the effect of *day* or *day^2* at the mean of vulnerability is depicted.

ΔR^2, change in R^2 for model term; *b*, unstandardized beta; LV, less vulnerable; MV, more vulnerable; *SEM*, standard error of the mean.

[a]Factor composed of median reaction time for correct responses on the Visual Object Learning Task [3] and median reaction time for hits on the Fractal 2-Back [6] (working memory), mean excess clicks and median reaction time for correct responses on the Line Orientation Task (visuospatial processing) [4], and throughput (number correct/minute) and median RT for correct responses on the Digit Symbol Substitution Task (processing speed) [5].

[b]Factor composed of number of true positives (hits) on the Visual Object Learning Task [3] and sensitivity (number of true positives / images warranting screen tap) on the Fractal 2-Back [6] (working memory).

[c]Factor composed of number of correct responses and mean rotation error on the Line Orientation Task (visuospatial processing) [4].

[§]Model includes *time of day*.

**p* < .05; ***p* < .01; ****p* < .001, two-tailed.

Table S4. Comparisons of cognitive factor performance during sleep restriction (SR) and recovery (REC) days to baseline (BL).

Outcome	Level	BL[a] (ref)	SR: Difference (*SEM*)					REC: Difference (*SEM*)	
			1	2	3	4	5	1	2
Speed and efficiency[b§]	*LV*	69.18 (1.81)	**2.07 (.81)**	**3.06 (.80)**	**3.64 (.81)**	**2.83 (.89)**	**4.41 (.94)**	**5.83 (1.09)**	**6.57 (1.44)**
Recall[c]	*LV*	77.83 (4.18)	.32 (2.00)	2.39 (1.99)	1.36 (2.00)	−1.68 (2.22)	***4.25 (2.33)***	**5.33 (2.71)**	**9.47 (3.54)**
Visuospatial accuracy[d]	*Mean*	79.44 (1.53)	−.71 (.99)	**−2.15 (.98)**	***−1.87 (.98)***	**−3.02 (1.09)**	−.94 (1.15)	.18 (1.34)	−.13 (1.68)

Notes: All factors scaled from 0 (worst performance) to 100 (best performance). Vulnerability calculated as difference between within-person mean of psychomotor vigilance task (PVT) lapses on last sleep restriction day versus last baseline day, per person (see Fig. 6 for vulnerability level per participant). For significant *day* or *day*^{2}**attentional vulnerability* interactions, comparisons to baseline reference point (BL; day succeeding third BL night) were made at lower vulnerability (LV, less vulnerable) and at higher vulnerability (MV, more vulnerable) levels. LV estimates were obtained by re-centering attentional vulnerability at 1 standard deviation (*SD*) below the mean. MV estimates were obtained by re-centering attentional vulnerability at 1 *SD* above the mean (*N* = 15 in all analyses; refer to Statistical Analyses). For non-significant interactions, comparisons to BL are made at the mean of vulnerability. Comparisons to BL only made for outcomes demonstrating significant trajectories across study (see Table S3, "By Vulnerability").

BL, baseline; LV, more vulnerable; MV, more vulnerable; REC, recovery; *SEM*, standard error of the mean; SR, sleep restriction.

[a]Last baseline day (succeeding third baseline night).

[b]Factor composed of median reaction time for correct responses on the Visual Object Learning Task [3] and median reaction time for hits on the Fractal 2-Back [6] (working memory), mean excess clicks and median reaction time for correct responses on the Line Orientation Task (visuospatial processing) [4], and throughput (number correct/minute) and median RT for correct responses on the Digit Symbol Substitution Task (processing speed) [5].

[c]Factor composed of number of true positives (hits) on the Visual Object Learning Task [3] and sensitivity (number of true positives / images warranting screen tap) on the Fractal 2-Back [6] (working memory).

[d]Factor composed of number of correct responses and mean rotation error on the Line Orientation Task (visuospatial processing) [4].

[§]Model includes *time of day*.

Bold values indicate SR or REC day was significantly different from BL (*p* < .05). ***Bold italic*** values indicate SR or REC day was marginally different from BL (*p* < .10).

Table S5. Trajectory of PVT, VOLT, F2B, LOT, and DSST performance across baseline days.

Outcome	Mean (*SEM*)			*b (SEM)*	Pairwise comparisons *b* (*SEM*)		
	BL1	BL2	BL3		2 vs 1	3 vs 1	3 vs 2
**PVT (attention)**							
Lapses[§]	4.37 (1.39)	3.96 (1.45)	4.62 (1.39)	.13 (.33)	---	---	---
**VOLT (working memory)**							
Correct responses	17.81 (.46)	17.66 (.48)	17.60 (.45)	−.10 (.11)	---	---	---
Hits	8.73 (.24)	8.72 (.27)	8.59 (.24)	−.07 (.08)	---	---	---
Correct rejections	9.08 (.32)	8.93 (.34)	9.01 (.32)	−.03 (.06)	---	---	---
Median RT, correct (ms)[‖]	1174.67 (40.07)	1049.14 (43.36)	954.20 (39.93)	−110.17*** (11.58)	−125.53*** (28.64)	−220.47*** (23.15)	−94.94** (28.45)
**F2B (working memory)**							
Accuracy[a]	88.58 (1.17)	88.91 (1.26)	88.93 (1.17)	.17 (.33)	---	---	---
Sensitivity[a]	66.75 (2.92)	70.02 (3.24)	69.49 (2.90)	1.36 (.97)	---	---	---
Specificity[a§]	95.55 (1.12)	94.93 (1.20)	95.13 (1.12)	−.21 (.30)	---	---	---
Median RT, hits (ms)	559.14 (21.73)	541.40 (23.08)	552.59 (21.66)	−3.21 (5.40)	---	---	---
**LOT (visuospatial processing)**							
Correct responses	13.81 (.56)	13.58 (.63)	13.96 (.56)	.08 (.19)	---	---	---
Rotation error[b] (*M*)	.46 (.04)	.48 (.04)	.47 (.04)	.01 (.01)	---	---	---
Excess clicks, correct (*M*)[‖]	2.81 (.38)	2.35 (.39)	1.98 (.37)	−.41*** (.07)	−.46* (.18)	−.83*** (.15)	−.37* (.18)
Median RT, correct (s)[§#]	5.41 (.29)	4.93 (.31)	4.54 (.29)	−.43*** (.08)	−.47* (.19)	−.87*** (.16)	−.39* (.19)
**DSST (processing speed)**							
Throughput[c§#]	57.98 (1.72)	58.75 (1.75)	59.47 (1.71)	.74** (.26)	.77 (.61)	1.49** (.51)	.71 (.60)
Median RT, correct (ms)[§]	819.17 (30.24)	823.09 (30.85)	811.50 (30.21)	−3.92 (4.62)	---	---	---

b, unstandardized beta; BL, baseline; DSST, Digit Symbol Substitution Task; F2B, Fractal 2-Back; LOT, Line Orientation Task; *M,* mean; PVT, psychomotor vigilance task; RT, reaction time; s, seconds; *SEM*, standard error of the mean; VOLT, Visual Object Learning Task.

[a]Ranges from 0 (minimally accurate, sensitive, or specific) to 100 (maximally accurate, sensitive, or specific).

[b]Ranges from 0 (no rotation error) to 30 (perpendicular line; highest rotation error).

[c]Number correct per minute.

df for pairwise tests: [‖]*df* = 207; [#]*df* = 206.

[§]Model includes *time of day*.

*$p < .05$, **$p < .01$, ***$p < .001$, two-tailed.

Table S6. Factor loadings for cognitive variables in principal factor analysis.

Cognitive variable	Factor name: Speed and efficiency	Recall	Visuospatial accuracy
**VOLT**			
Hits	−.12	**.28**	.05
Median RT, correct (ms)	**.50**	−.27	.10
**F2B**			
Sensitivity[a]	.15	**.21**	.11
Median RT, hits (ms)	**.28**	.06	.02
**LOT**			
Correct responses	.12	.32	**.51**
Rotation error (*M*)[b]	−.05	.45	**.50**
Excess clicks, correct (*M*)	**.62**	−.46	.17
Median RT correct (s)	**.69**	−.38	.18
**DSST**			
Throughput[c]	**.66**	.53	−.19
Median RT, correct (ms)	**.62**	.48	−.32

Notes: Principal factor analysis (PFA) was conducted (method=principal) with oblique rotation (rotate=promax) [8]. Visual Object Learning Task correct rejections and Fractal 2-Back specificity had low final communality estimates (less than 1% of the total communality) and were removed from the PFA. **Bold** values indicate variable loaded most highly onto that factor.

DSST, DSST, Digit Symbol Substitution Task (processing speed) [5]; F2B, Fractal 2-Back (working memory) [6]; LOT, Line Orientation Task (visuospatial processing) [4]; *M*, mean; RT, reaction time; s, seconds; VOLT, Visual Object Learning Task (working memory) [3].

[a]Range: 0 (minimally sensitive) to 100 (maximally sensitive).

[b]Range: 0 (no rotation error) to 30 (perpendicular line; highest rotation error).

[c]Number correct per minute.
